# Prognostic value of the MDACC–NLR score in extensive-stage small-cell lung cancer treated with first-line chemoimmunotherapy

**DOI:** 10.3389/fimmu.2025.1681658

**Published:** 2025-11-07

**Authors:** Dan Li, Xiaolin Li, Ning Liu, Bo Wang, Hui Jin, Yan Liu, Jiayin Liu, Xue Zhang, Long Wang, Zhisong Fan, Li Feng, Jing Han, Jing Zuo, Yudong Wang

**Affiliations:** Department of Medical Oncology, The Fourth Hospital of Hebei Medical University, Shijiazhuang, China

**Keywords:** extensive-stage small cell lung cancer (ES-SCLC), chemoimmunotherapy, prognostic scoring model, MDACC-NLR, survival prediction

## Abstract

**Objective:**

To evaluate the prognostic performance of six scoring systems in predicting outcomes of first-line chemo-immunotherapy in patients with extensive-stage small cell lung cancer (ES-SCLC), aiming to guide individualized treatment.

**Methods:**

This single-center retrospective study included 197 ES-SCLC patients treated with first-line chemo-immunotherapy. Clinical and laboratory data were collected, including baseline characteristics, treatment responses, and survival outcomes. The prognostic impact of six scoring systems (RHM, MDACC, MDACC+NLR, MDA-ICI, LIPI, GRIm) was assessed using univariate and multivariate Cox regression analyses for progression-free survival (PFS) and overall survival (OS). Kaplan–Meier analysis was conducted for risk stratification.

**Results:**

By the last follow-up (October 15, 2024), the median follow-up was 12 months, with 113 deaths (57.3%). The objective response rate was 75.6%. ECOG ≥1, lung metastasis, and liver metastasis were independent predictors of poorer PFS and OS. Among the scoring systems, only MDACC+NLR effectively stratified patients: low-risk patients had significantly longer PFS and OS (both p = 0.02). MDACC alone did not distinguish PFS among risk groups (p = 0.17) but showed significant OS differences (p = 0.02). Other systems (RHM, MDA-ICI, LIPI, GRIm) lacked significant discriminatory ability for both PFS and OS (all p > 0.05).

**Conclusion:**

ECOG ≥1, lung metastasis, and liver metastasis are adverse prognostic factors for ES-SCLC patients receiving first-line chemo-immunotherapy. The MDACC+NLR scoring system provides superior predictive value for treatment outcomes and survival, supporting its potential utility for clinical risk stratification.

## 1 Introduction

According to the latest data released in 2025 by the International Agency for Research on Cancer (IARC), a specialized agency of the World Health Organization, lung cancer remains the leading cause of cancer incidence and mortality worldwide. It has ranked first in global cancer-related deaths for ten consecutive years. Among all lung cancer subtypes, small cell lung cancer (SCLC) accounts for a significant proportion of lung cancer–related deaths (1). In China, data from the National Cancer Center reported more than 1.06 million new lung cancer cases and over 730,000 deaths in 2022. Lung cancer continues to be the most common and deadliest malignancy in the country, posing a serious threat to public health (2).

Small cell lung cancer (SCLC) is a highly aggressive subtype of lung cancer (3), accounting for approximately 13% to 15% of all lung cancer cases (4). Its occurrence is strongly associated with tobacco smoking (5), SCLC is characterized by rapid growth, early dissemination, and a high propensity for recurrence and metastasis. Recent reviews have highlighted the molecular heterogeneity of SCLC and its implications for therapeutic response and drug development. Distinct transcriptional subtypes such as ASCL1, NEUROD1, POU2F3, and YAP1 not only exhibit unique biological behaviors but may also influence sensitivity to immunotherapy and targeted agents” (6). Although initially sensitive to chemotherapy and radiotherapy, the disease frequently relapses and has a poor overall prognosis. Clinically, SCLC is classified into limited-stage (LS) and extensive-stage (ES) disease. The 5-year survival rate for LS-SCLC is approximately 30%, while for ES-SCLC it is only around 3%. The median survival time is about 15–20 months for LS patients and 8–13 months for those with ES-SCLC (7). Despite a recent decline in the overall incidence of lung cancer, the mortality rate of SCLC remains alarmingly high. Due to the lack of effective screening methods, most patients are diagnosed at an advanced stage, presenting significant therapeutic challenges.

Over the past few decades, platinum-based doublet chemotherapy has been the standard first-line treatment for small cell lung cancer (SCLC). It usually consists of a platinum agent combined with etoposide or irinotecan. However, most patients experience disease recurrence within four months after completing initial therapy.

In recent years, immunotherapy—particularly immune checkpoint inhibitors (ICIs)—has become a major breakthrough in cancer treatment (8). In extensive-stage SCLC (ES-SCLC), combining immunotherapy with chemotherapy has significantly improved progression-free survival (PFS) compared with chemotherapy alone. As a result, chemoimmunotherapy (CIT) is now recommended as the standard first-line regimen for ES-SCLC (9).

Despite CIT being established as the first-line standard of care and demonstrating survival benefits in several clinical trials (10, 11), not all patients derive equal benefit from this approach. Most existing studies on first-line treatment for ES-SCLC are based on rigorously designed randomized controlled trials (RCTs). However, due to the strict inclusion criteria of RCTs, their findings may have limited generalizability to real-world patient populations. In contrast, real-world evidence provides a more comprehensive picture of the effectiveness and survival outcomes associated with CIT in clinical practice.

Currently, there are no reliable or validated biomarkers that can accurately predict the efficacy or prognosis of chemoimmunotherapy (CIT) in ES-SCLC. To address this gap, several large-scale clinical studies have developed prognostic scoring systems based on clinical risk factors. These include the Royal Marsden Hospital Index (RMH), the MD Anderson Clinical Center Score (MDACC), the MDACC score combined with the neutrophil-to-lymphocyte ratio (MDACC+NLR), the MD Anderson Immune Checkpoint Inhibitor Score (MDA-ICI), the Lung Immune Prognostic Index (LIPI), and the Gustave Roussy Immune Score (GRIm) (7, 12–14). These scoring systems were primarily designed to identify patients with malignant tumors who may derive greater benefit from novel therapies in clinical trials and have also demonstrated value in prognostic stratification. However, the predictive and prognostic utility of these scoring systems in the context of first-line CIT for ES-SCLC remains unclear and requires further investigation.

In this study, we retrospectively analyzed clinicopathological characteristics and survival data from 197 patients with ES-SCLC who received first-line chemoimmunotherapy (CIT) in a real-world setting. We evaluated the prognostic value and predictive performance of six established scoring systems—RHM, MDACC, MDACC+NLR, MDA-ICI, LIPI, and GRIm—in the context of first-line CIT. This study aimed to identify the most effective prognostic model for stratifying ES-SCLC patients and guiding treatment decisions. Our findings may provide important insights for selecting patients most likely to benefit from CIT and offer new evidence to support the development of personalized treatment strategies for ES-SCLC.

## 2 Materials and methods

### 2.1 Study population

#### 2.1.1 Patient selection and treatment details

**Figure 1:**
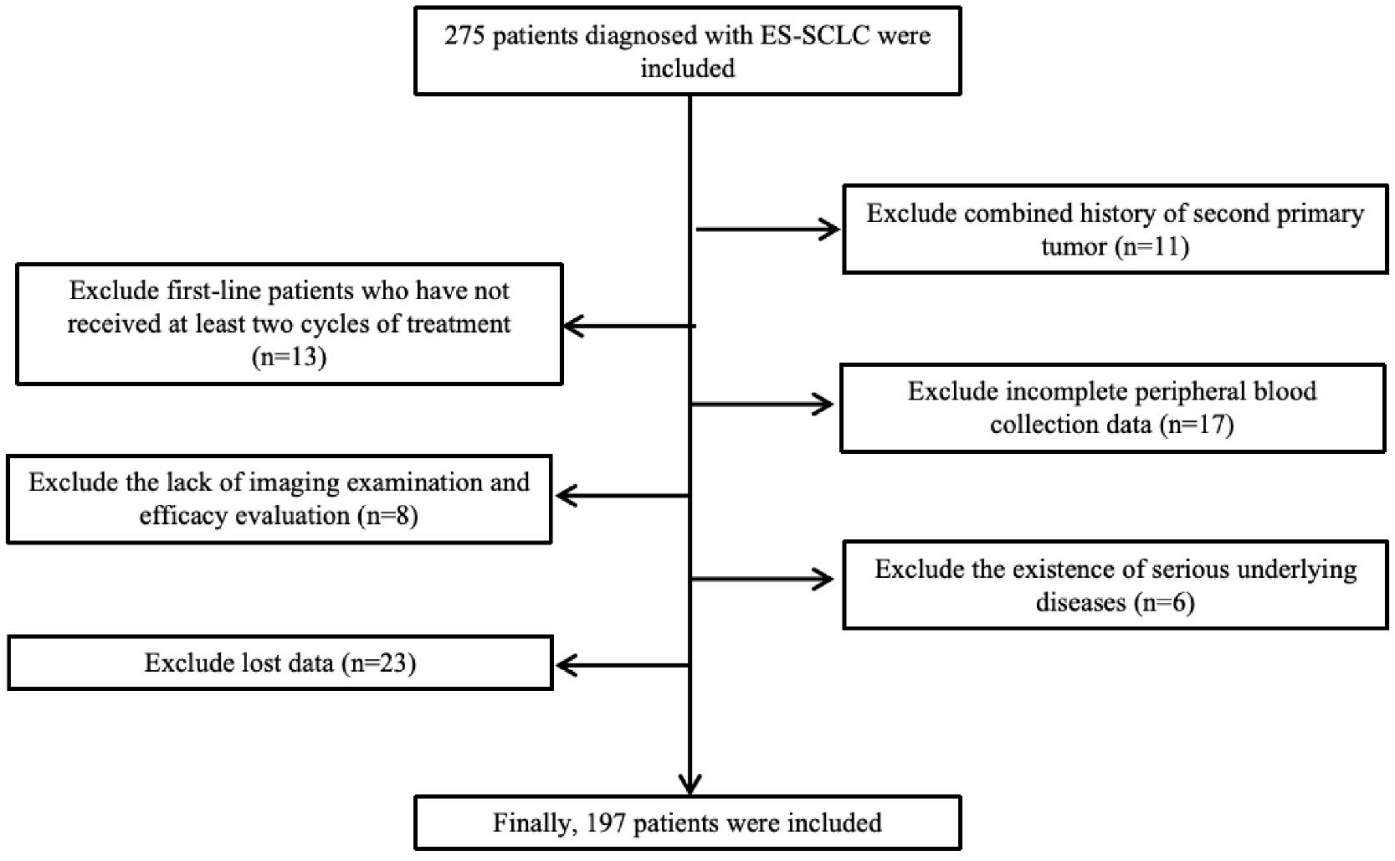
Flow diagram of patient enrollment. A total of 275 patients diagnosed with ES-SCLC were screened; 78 were excluded for predefined reasons, and 197 patients were finally included in the analysis.

This retrospective study reviewed 275 patients diagnosed with extensive-stage small cell lung cancer (ES-SCLC) at our institution between December 2016 and June 2024.Eligible patients met the following inclusion criteria (1): histologically or cytologically confirmed ES-SCLC (2); receipt of first-line platinum-based chemotherapy combined with a PD-1 or PD-L1 inhibitor; and (3) availability of complete baseline clinical and laboratory data. Patients were excluded if they (1) had concurrent malignancies (2); lacked complete follow-up or treatment information; or (3) were lost to follow-up before the first efficacy assessment. After applying these criteria, 197 patients were included in the final analysis. A detailed summary of inclusion and exclusion criteria is presented below, and the patient selection process is depicted in **Figure 1**.

### 2.2 Inclusion and exclusion criteria

#### 2.2.1 Inclusion criteria

Patients were eligible for inclusion if they met the following criteria:

1. Pathologically confirmed SCLC according to WHO criteria;
2. Extensive-stage disease defined by VALSG classification;
3. Age ≥18 years;
4. No contraindications to systemic therapy based on baseline evaluations;
5. Received ≥2 cycles of first-line platinum-based chemoimmunotherapy with evaluable efficacy data;
6. Tumor response assessed by CT, MRI, bone scan, or PET-CT;
7. Availability of complete baseline clinical, laboratory, and follow-up data.

#### 2.2.2 Exclusion criteria

Patients were excluded based on the following criteria:

1. Receipt of any prior antitumor therapy (e.g., radiotherapy or surgery) before first-line treatment;
2. History of another primary malignancy;
3. Lack of imaging data or unassessable tumor response;
4. Presence of active infection, severe autoimmune disease, or major organ dysfunction;
5. Incomplete or missing baseline clinical or follow-up information.

### 2.3 Study design

#### 2.3.1 Treatment regimens

All enrolled patients with ES-SCLC were categorized into two groups: the chemoimmunotherapy (CIT) group and the chemotherapy (CT) group. Chemotherapy agents included etoposide, either alone or in combination with cisplatin, carboplatin, or lobaplatin. Immunotherapy agents included:

• Atezolizumab (Roche Diagnostics GmbH, 1200 mg every 3 weeks)
• Durvalumab (AstraZeneca UK Limited, 1500 mg every 3 weeks)
• Serplulimab (Shanghai Henlius Biotech Inc., 4.5 mg/kg every 3 weeks)
• Tislelizumab (BeiGene, Guangzhou, 200 mg every 3 weeks)
• Toripalimab (Zhonghe Biopharma, Suzhou, 3 mg/kg every 3 weeks)
• Adebrelimab (Hengrui Medicine Co., Ltd., Jiangsu, 20 mg/kg every 3 weeks)

Chemotherapy was administered for 2–6 cycles. Patients then either continued with maintenance therapy or discontinued treatment upon disease progression, unacceptable toxicity, or death.

#### 2.3.2 Peripheral blood biomarkers

Baseline peripheral blood parameters within one week prior to initiation of CIT were extracted from the hospital electronic medical record system. These included white blood cell count (WBC), absolute neutrophil count (ANC), absolute lymphocyte count (ALC), platelet count (PLT), serum lactate dehydrogenase (LDH), and serum albumin (ALB).

### 2.4 Derived inflammatory indices

1. Neutrophil-to-lymphocyte ratio (NLR) was calculated as: NLR = ANC/ALC
2. Derived neutrophil-to-lymphocyte ratio (dNLR) was calculated as: dNLR = ANC/(WBC − ANC)

### 2.5 Response evaluation criteria

Tumor response was assessed according to the Response Evaluation Criteria in Solid Tumors (RECIST), version 1.1:

Complete Response (CR): Disappearance of all target lesions, no new lesions, normalization of tumor markers, and sustained response for at least 4 weeks.

Partial Response (PR): At least a 30% reduction in the sum of the diameters of target lesions compared to baseline, with no new lesions, sustained for at least 4 weeks.

Stable Disease (SD): Disease status between PR and PD, with no new lesions, maintained for 6–8 weeks.

Progressive Disease (PD): A ≥20% increase in the sum of diameters of target lesions from the smallest value recorded or the appearance of one or more new lesions.

The Objective Response Rate (ORR) was calculated as: ORR = (CR + PR)/total number of patients × 100%.

### 2.6 Prognostic scoring systems

Detailed scoring criteria and grouping rules for the six prognostic systems (RMH Score (15), MDACC Score (16), MDACC+NLR Score (11), MDA-ICI Score (13), Definition of LIPI Score (7), GRIm Score (14)) are listed in **Appendix Table 1**.

### 2.7 Follow-up

Patient follow-up was conducted using the institutional follow-up center database, as well as the outpatient and inpatient medical record systems. For patients with incomplete follow-up information in the system, additional data were collected via telephone interviews. The final date of follow-up was October 15, 2024.

### 2.8 Study endpoints

The primary endpoints of this study were overall survival (OS) and progression-free survival (PFS).

OS was defined as the time interval from the initial diagnosis of ES-SCLC to death from any cause or last follow-up.

PFS was defined as the time from initial diagnosis to the date of disease progression or death from any cause.

Patients who had not experienced disease progression or death by the end of follow-up were censored at the date of their last follow-up.

### 2.9 Statistical analysis

All statistical analyses were conducted using SPSS software, version 25.0. Categorical variables were presented as frequencies and percentages. Group differences were assessed using the chi-square test. Survival comparisons between groups were performed using Kaplan–Meier survival curves, with statistical significance evaluated by the log-rank test. Prognostic factors were identified using Cox proportional hazards regression models, and adjusted hazard ratios (HRs) with 95% confidence intervals (CIs) were calculated. All tests were two-sided, and p < 0.05 was considered statistically significant.

A Cox proportional hazards model using the MDACC+NLR composite score as the predictor was applied to evaluate its prognostic value for overall survival (OS). Model performance was assessed by Harrell’s concordance index (C-index, 1,000 bootstrap resamples) and bias-corrected calibration curves at 6 and 12 months. A nomogram was constructed to visualize individualized survival probabilities, and decision curve analysis (DCA) was used to estimate the clinical net benefit across threshold probabilities. All analyses were performed in R (version 4.x) using the rms, Hmisc, rmda, and timeROC packages, with a two-sided α = 0.05.

## 3 Results

### 3.1 Patient enrollment and data collection

A total of 275 patients diagnosed with extensive-stage small cell lung cancer (ES-SCLC) who received first-line chemoimmunotherapy (CIT) were initially screened. After excluding 78 patients due to receipt of fewer than two treatment cycles, history of another primary malignancy, incomplete peripheral blood data, severe comorbidities, or loss to follow-up, 197 patients with complete clinical and follow-up data were included in the final analysis. As of the last follow-up on October 15, 2024, the median follow-up time was 16 months (95% CI: 14.7–17.3), and 113 deaths had occurred.

### 3.2 Clinicopathological characteristics

Among the 197 patients, 160 (81.8%) were male and 37 (18.2%) were female. The median age was 65 years; 101 patients (51.3%) were <65 years, and 96 (48.7%) were ≥65 years. Forty-five patients (22.8%) had an ECOG performance status <1, while 152 (77.2%) had ECOG ≥1.

At diagnosis, metastases were observed in the brain (58 patients, 29.4%), liver (72, 36.5%), lung (26, 13.2%), bone (140, 71.1%), and adrenal glands (33, 16.8%). Multi-organ metastases (≥2 organs) were present in 161 patients (81.7%). Forty-seven (23.9%) underwent brain radiotherapy, and 12 (6.1%) received prophylactic cranial irradiation (PCI). A smoking history was reported in 104 patients (52.8%), while 93 (47.2%) were non-smokers (**Table 1**).

**Table 1:** Baseline characteristics of included patients (n=197).

Characteristics	n	%	Characteristics	n	%
Sex			Lung metastasis		
Male	160	81.8	Yes	26	13.2
Female	37	18.2	No	171	86.8
Age			Liver metastasis		
≥65	96	48.7	Yes	72	36.5
<65	101	51.3	No	125	63.6
ECOG			Adrenal metastasis		
0	45	22.8	Yes	33	16.8
1-2	152	77.2	No	164	83.2
Smoking status			Metastatic sites		
Smoker	134	68.0	Yes	161	81.7
Non-smoker	63	32.0	No	36	18.3
Alcohol consumption			Thoracic radiotherapy		
Drinking	104	52.8	Yes	47	23.9
Non-drinking	95	47.2	No	150	76.1
Brain metastasis			Brain radiotherapy		
Yes	58	29.4	Yes	31	15.7
No	139	70.6	No	166	84.3
Bone metastasis			PCI		
Yes	57	28.9	Yes	12	6.1
No	140	71.1	No	185	93.9

ECOG, Eastern Cooperative Oncology Group performance status; PCI, Prophylactic Cranial Irradiation.

### 3.3 Prognostic impact of clinicopathological characteristics

#### 3.3.1 Progression-free survival

In univariate Cox regression, ECOG <1 was associated with longer PFS (HR: 0.60, 95% CI: 0.38–0.95; P = 0.02). Lung metastases (HR: 0.49, 95% CI: 0.31–0.76; P < 0.01) and liver metastases (HR: 0.65, 95% CI: 0.47–0.93; P = 0.02) were associated with shorter PFS. Other factors showed no significant association with PFS (P > 0.05). Multivariate analysis confirmed ECOG ≥1 (HR: 0.57, 95% CI: 0.36–0.91; P = 0.01), lung metastases (HR: 0.45, 95% CI: 0.28–0.73; P < 0.01), and liver metastases (HR: 0.67, 95% CI: 0.47–0.94; P = 0.02) as independent negative predictors of PFS (**Table 2**).

**Table 2:** Univariate and multivariate Cox regression analyses of clinicopathological characteristics associated with progression-free survival (PFS) in ES-SCLC patients receiving first-line chemoimmunotherapy.

Variable	Univariate analysis (PFS)		Multivariate analysis (PFS)	
	HR (95%CI)	*P*	HR (95%CI)	*P*
Gender (Female vs Male)	0.88 (0.57-1.34)	0.56		
Age				
≥65 vs <65	0.91 (0.65-1.28)	0.61		
≥52 vs <52*	0.80 (0.43-1.49)	0.48		
ECOG (0 vs 1-2)	0.60 (0.38-0.94)	0.02	0.57 (0.36-0.91)	0.01
Smoking status (no vs yes)	0.85 (0.59-1.22)	0.39		
Brain metastasis (metastasis vs no metastasis)	0.79 (0.54-1.14)	0.21		
Bone metastasis (no metastasis vs metastasis)	0.83 (0.57-1.18)	0.31		
Lung metastasis (no metastasis vs metastasis)	0.49 (0.31-0.79)	<0.01	0.45 (0.28-0.73)	<0.01
Liver metastasis (no metastasis vs metastasis)	0.66 (0.47-0.93)	0.02	0.67 (0.47-0.94)	0.02
Adrenal metastasis (no metastasis vs metastasis)	0.74 (0.48-1.14)	0.18	0.71 (0.45-1.08)	**0.11**
Metastatic sites (≤2 vs >2)	0.84 (0.54-1.29)	0.43		
Thoracic radiotherapy (yes vs no)	0.98 (0.67-1.43)	0.94		
Brain radiotherapy (no vs yes)	0.81 (0.53-1.22)	0.32		
PCI (yes vs no)	0.92 (0.48-1.75)	0.80		
ANC (>4.9 vs ≤4.9)	0.81 (0.58-1.13)	0.22		
ALC (<1.8 vs ≥1.8)	0.98 (0.68-1.41)	0.92		
PLT (>300 vs <300)	0.85 (0.58-1.23)	0.39		
LDH (120-250 vs >250)	0.91 (0.65-1.28)	0.61		
ALB (>35 vs ≤35)	0.92 (0.57-1.48)	0.73		
NLR (≤6 vs >6)	0.98 (0.57-1.68)	0.96		
dNLR (>3 vs ≤3)	0.97 (0.65-1.43)	0.86		

PCI, Prophylactic Cranial Irradiation; ANC, Absolute Neutrophil Count; ALC, Absolute Lymphocyte Count; PLT, Platelet Count; LDH, Lactate Dehydrogenase; ALB, Albumin; NLR, Neutrophil-to-Lymphocyte Ratio; dNL, Derived Neutrophil-to-Lymphocyte Ratio. *Age groups in the MDA-ICI scoring system.

The bold values indicate statistically significant results (P < 0.05).

#### 3.3.2 Overall survival

Univariate analysis showed liver metastases (HR: 0.54, 95% CI: 0.37–0.79; P < 0.01) and lung metastases (HR: 0.54, 95% CI: 0.32–0.92; P = 0.02) were associated with worse OS. ECOG <1 trended toward longer OS (HR: 0.58, 95% CI: 0.34–1.01; P = 0.06), though not statistically significant. In multivariate analysis, ECOG <1 (HR: 0.56, 95% CI: 0.32–1.00; *P* = 0.05), lung metastases (HR: 0.56, 95% CI: 0.32–0.97; *P* = 0.04), and liver metastases (HR: 0.64, 95% CI: 0.42–0.98; *P* = 0.04) remained independent predictors of poorer OS (**Table 3**).

**Table 3:** Univariate and multivariate Cox regression analyses of clinicopathological characteristics associated with overall survival (OS) in ES-SCLC patients receiving first-line chemoimmunotherapy.

Variable	Univariate analysis (0S)		Multivariate analysis (0S)	
	HR (95%CI)	*P*	HR (95%CI)	*P*
Gender (Female vs Male)	0.88 (0.57-1.34)	0.42		
Age				
≥65 vs <65	0.78 (0.54-1.13)	0.2		
≥52 vs <52*	0.71 (0.36-1.46)	0.35		
ECOG (0 vs 1-2)	0.58 (0.34-1.01)	0.06	0.56 (0.32-1.00)	0.05
Smoking status (no vs yes)	0.75 (0.51-1.13)	0.17	0.85 (0.56-1.29)	0.45
Brain metastasis (metastasis vs no metastasis)	0.63 (0.41-0.98)	0.04	0.70 (0.43-1.13)	0.15
Bone metastasis (no metastasis vs metastasis)	0.68 (0.46-1.01)	0.06	0.81 (0.53-1.24)	0.34
Lung metastasis (no metastasis vs metastasis)	0.54 (0.32-0.88)	0.02	0.56 (0.32-0.97)	0.04
Liver metastasis (no metastasis vs metastasis)	0.54 (0.37-0.79)	<0.01	0.64 (0.42-0.98)	0.04
Adrenal metastasis (no metastasis vs metastasis)	0.73 (0.46-1.17)	0.20		
Metastatic sites (≤2 vs >2)	0.65 (0.41-1.03)	0.07	0.76 (0.41-1.39)	0.38
Thoracic radiotherapy (yes vs no)	0.73 (0.48-1.13)	0.16	0.88 (0.56-1.39)	0.61
Brain radiotherapy (no vs yes)	0.77 (0.47-1.25)	0.30		
PCI (yes vs no)	0.67 (0.29-0.54)	0.35		
ANC (>4.9 vs ≤4.9)	0.80 (0.55-1.16)	0.24		
ALC (<1.8 vs ≥1.8)	0.97 (0.66-1.43)	0.89		
PLT (>300 vs <300)	0.90 (0.60-1.35)	0.62		
LDH (120-250 vs >250)	0.88 (0.61-1.28)	0.51		
ALB (>35 vs ≤35)	0.97 (0.57-1.66)	0.93		
NLR (≤6 vs >6)	0.93 (0.51-1.71)	0.83		
dNLR (>3 vs ≤3)	0.99 (0.64-1.53)	0.98		

PCI, Prophylactic Cranial Irradiation; ANC, Absolute Neutrophil Count; ALC, Absolute Lymphocyte Count; PLT, Platelet Count; LDH, Lactate Dehydrogenase; ALB, Albumin; NLR, Neutrophil-to-Lymphocyte Ratio; dNL, Derived Neutrophil-to-Lymphocyte Ratio. *Age groups in the MDA-ICI scoring system.

### 3.4 Prognostic value of scoring systems

#### 3.4.1 1RMH score

**Figure 2:**
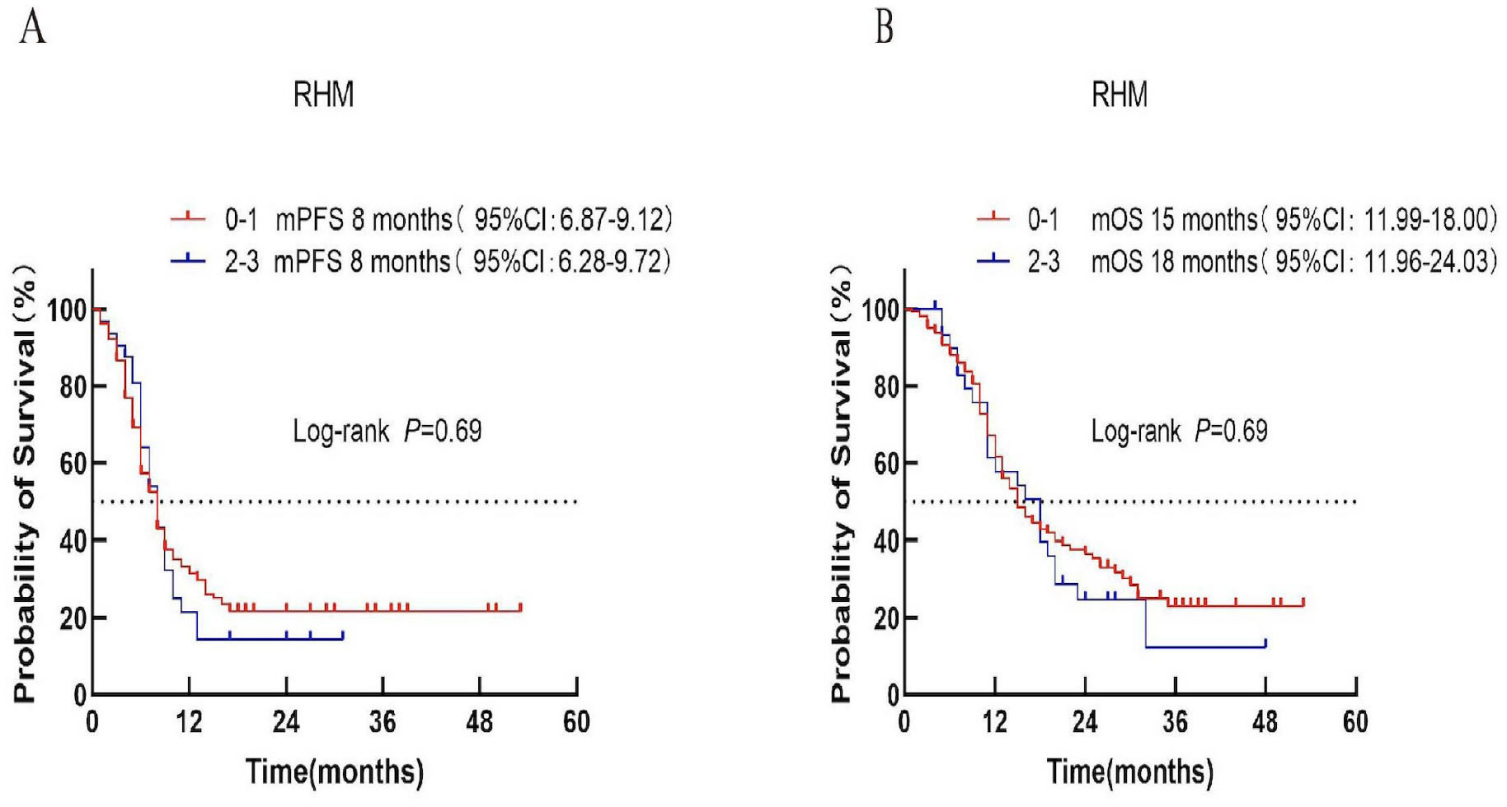
Survival analysis of the RMH scoring system in ES-SCLC patients treated with first-line chemoimmunotherapy. **(A)** Progression-free survival (PFS). **(B)** Overall survival (OS).

Patients were stratified into low-risk (score 0–1) and high-risk (score 2–3) categories according to the RMH score.Median PFS was 8 months in both groups (*P* = 0.69; **Figure 2A**). Median OS was 15 months for the low-risk group and 18 months for the high-risk group (*P* = 0.69; **Figure 2B**).

#### 3.4.2 MDACC score

**Figure 3:**
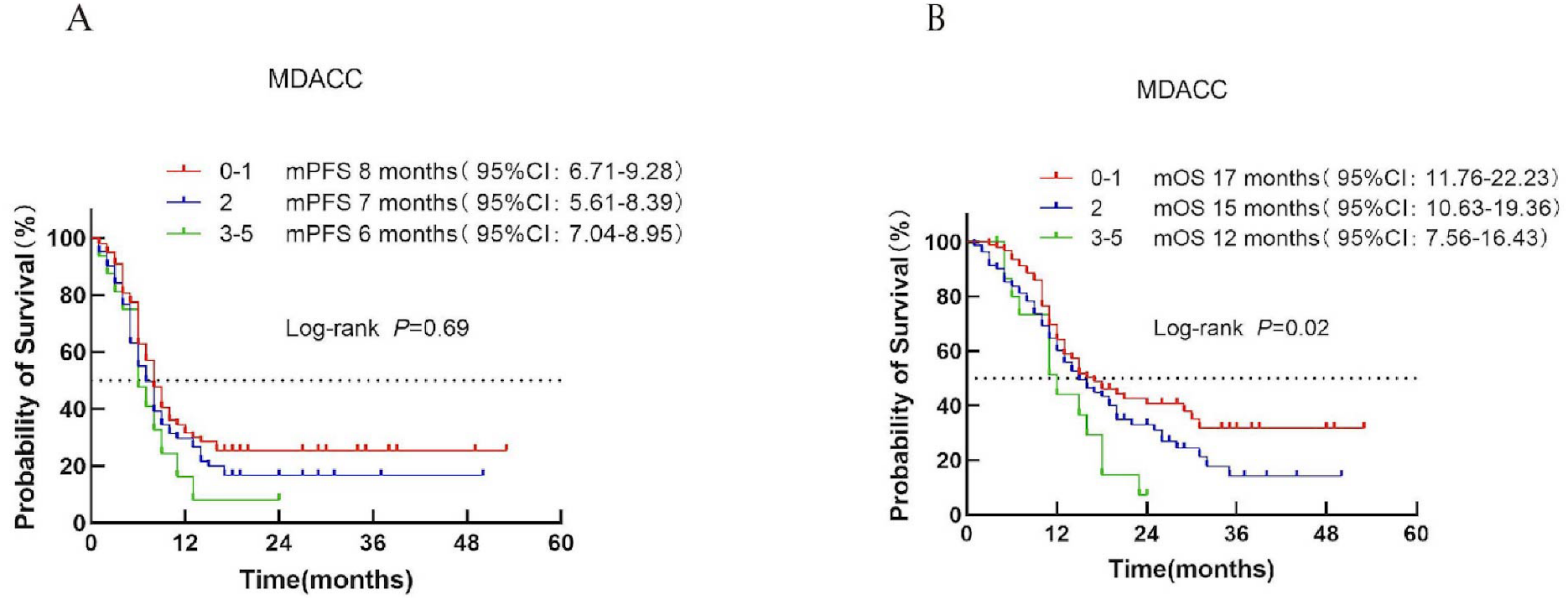
Survival analysis of the MDACC scoring system in ES-SCLC patients receiving first-line chemoimmunotherapy combined with immunotherapy. **(A)** PFS curves for each risk group. **(B)** OS curves with corresponding median survival times.

Based on the MDACC score, patients were divided into low-risk (score 0–1), intermediate-risk (score 2), and high-risk (score 3) categories. Median PFS was 8, 7, and 6 months for the respective groups (*P* = 0.69; **Figure 3A**). In contrast, median OS differed significantly among the three groups (17, 15, and 12 months; *P* = 0.02; **Figure 3B**).

#### 3.4.3 MDACC + NLR score

**Figure 4:**
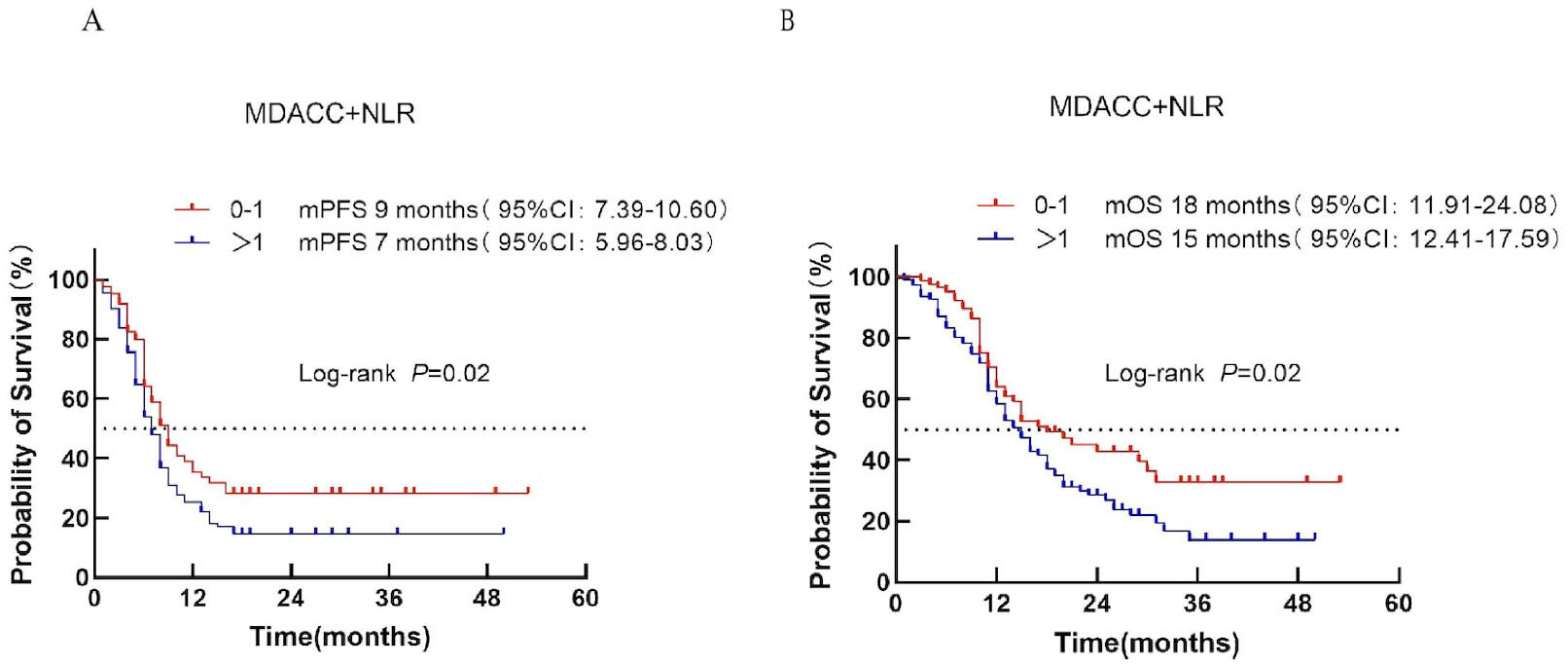
Survival analysis of the MDACC + NLR scoring system in ES-SCLC patients treated with first-line chemoimmunotherapy. **(A)** PFS. **(B)** OS.

Patients were stratified into low-risk (score 0–1) and high-risk (score >1) categories according to the MDACC + NLR score. Median PFS was 9 months in the low-risk group and 7 months in the high-risk group (*P* = 0.02; **Figure 4A**). Median OS was 18 vs. 15 months, respectively (*P* = 0.02; **Figure 4B**), demonstrating a clear prognostic distinction between the two groups.

#### 3.4.4 MDA-ICI score

**Figure 5:**
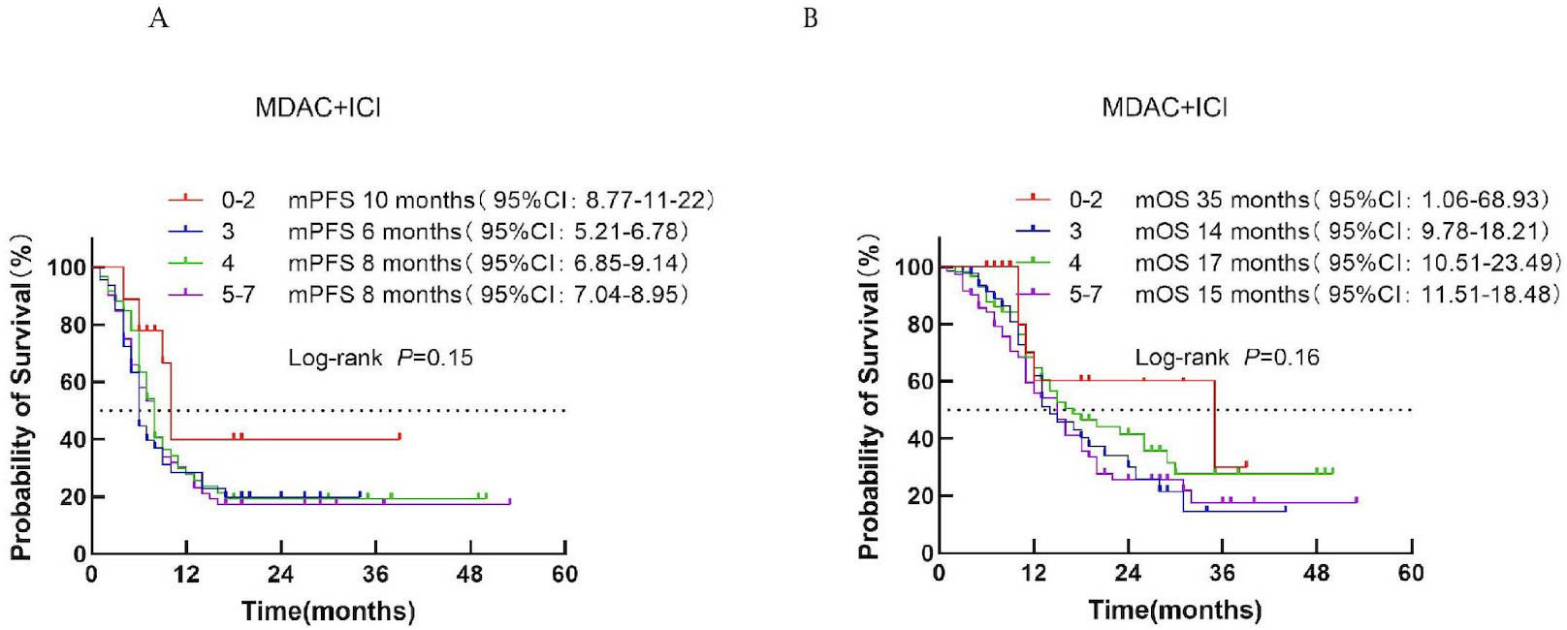
Survival analysis of the MDACC + ICI scoring system in ES-SCLC patients treated with first-line immunotherapy combination. **(A)** PFS. **(B)** OS.

According to the MDA-ICI score, patients were classified into three risk groups: low risk (score 0–2), intermediate risk (score 3–4), and high risk (score 5–7). Median PFS was 10, 7, and 8 months for the low-, intermediate-, and high-risk groups, respectively (*P* = 0.15; **Figure 5A**). Median OS was 35, 15, and 16 months, respectively (*P* = 0.16; **Figure 5B**). No significant survival difference was observed across risk categories, indicating limited prognostic value for this model.

#### 3.4.5 LIPI score

**Figure 6:**
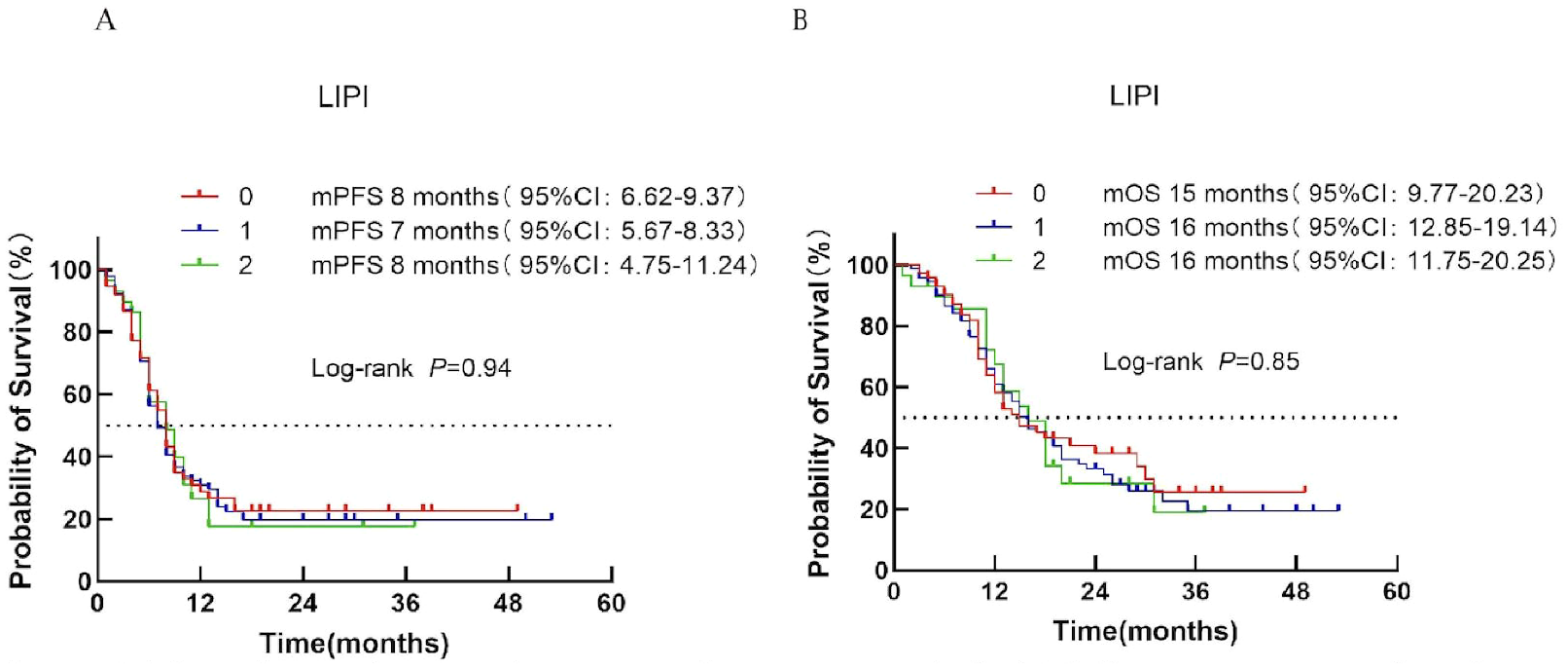
Survival analysis of the LIPI scoring system in ES-SCLC patients treated with first-line immunotherapy combination. **(A)** PFS. **(B)** OS.

Based on the Lung Immune Prognostic Index (LIPI), patients were classified into low-risk (score 0), intermediate-risk (score 1), and high-risk (score 2) groups corresponding to LIPI scores of 0, 1, and 2, respectively. Median PFS was 8, 7, and 8 months for the low-, intermediate-, and high-risk groups, respectively (*P* = 0.94; **Figure 6A**). Median OS was 15, 16, and 16 months, respectively (*P* = 0.85; **Figure 6B**).

#### 3.4.6 GRIm score

**Figure 7:**
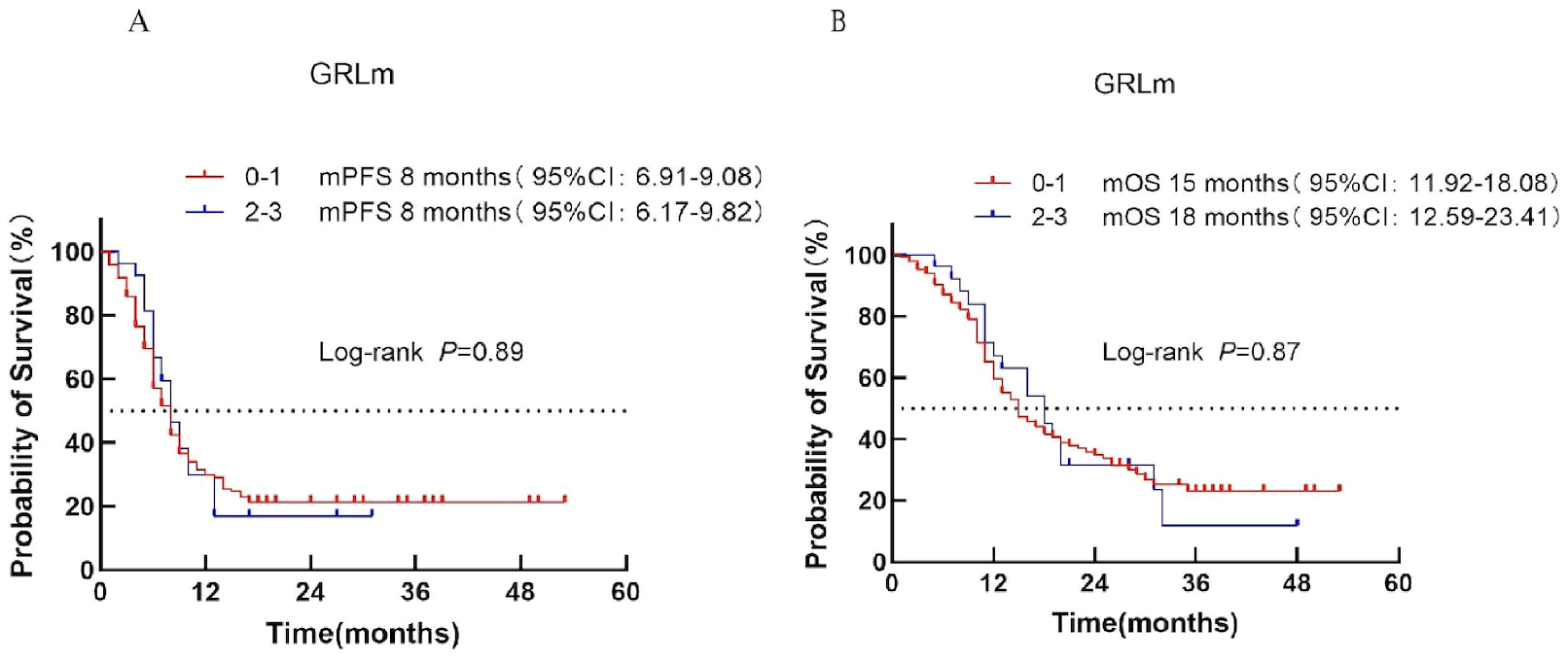
Survival analysis of the GRIm scoring system in ES-SCLC patients treated with first-line chemoimmunotherapy. **(A)** PFS. **(B)** OS.

Patients were divided into low-risk (score 0–1) and high-risk (score 2–3) categories according to the GRIm score. Median PFS was 8 months in both groups (*P* = 0.89; **Figure 7A**). Median OS was 15 months for the low-risk group and 18 months for the high-risk group (*P* = 0.87; **Figure 7B**), indicating no significant prognostic discrimination.

As shown in **Table 4**, the comparative analysis of six prognostic scoring systems revealed that only the MDACC+NLR composite score provided significant prognostic separation for both PFS and OS. The MDACC score showed moderate prognostic relevance for OS, whereas the RMH, MDA-ICI, LIPI, and GRIm models failed to demonstrate significant survival discrimination. These findings indicate that the MDACC+NLR model may serve as a practical and reliable prognostic tool for risk stratification in real-world ES-SCLC patients receiving chemoimmunotherapy.

**Table 4:** Progression-free survival (PFS) and overall survival (OS) according to six prognostic scoring systems.

Scoring system	Risk Classification	Median PFS (months, 95% CI)	P-value (PFS)	Median OS (months, 95% CI)	P-value (OS)
RMH Score	Low (0–1) vs High (2–3)	8 (6.9–9.1) vs 8 (6.7–9.2)	0.69	15 (13–17) vs 18 (15–20)	0.69
MDACC Score	Low (0–1)/Intermediate (2)/High (3-5)	8 (7–9)/7 (6–8)/6 (5–7)	0.69	17 (15–19)/15 (13–17)/12 (10–14)	**0.02**
MDACC+NLR Score	Low (0–1) vs High (>1)	9 (8–10) vs 7 (6–8)	**0.02**	18 (16–20) vs 15 (13–17)	**0.02**
MDA-ICI Score	Low (0–2)/Intermediate (3–4)/High (5–7)	10 (8–12)/7 (6–8)/8 (7–9)	0.15	35 (25–45)/15 (13–17)/16 (14–18)	0.16
LIPI Score	Low (0)/Intermediate (1)/High (2)	8 (7–9)/7 (6–8)/8 (7–9)	0.94	15 (13–17)/16 (14–18)/16 (14–18)	0.85
GRIm Score	Low (0–1) vs High (2–3)	8 (7–9) vs 8 (7–9)	0.89	15 (13–17) vs 18 (15–20)	0.87

RMH, Royal Marsden Hospital score; MDACC, MD Anderson Cancer Center score; NLR, neutrophil-to-lymphocyte ratio; MDA-ICI, MD Anderson–Immunotherapy score; LIPI, Lung Immune Prognostic Index; GRIm, Gustave Roussy Immune score; PFS, progression-free survival; OS, overall survival.

The bold values indicate statistically significant results (P < 0.05).

#### 3.4.7 The predictive ability of MDACC+NLR scoring system

**Figure 8:**
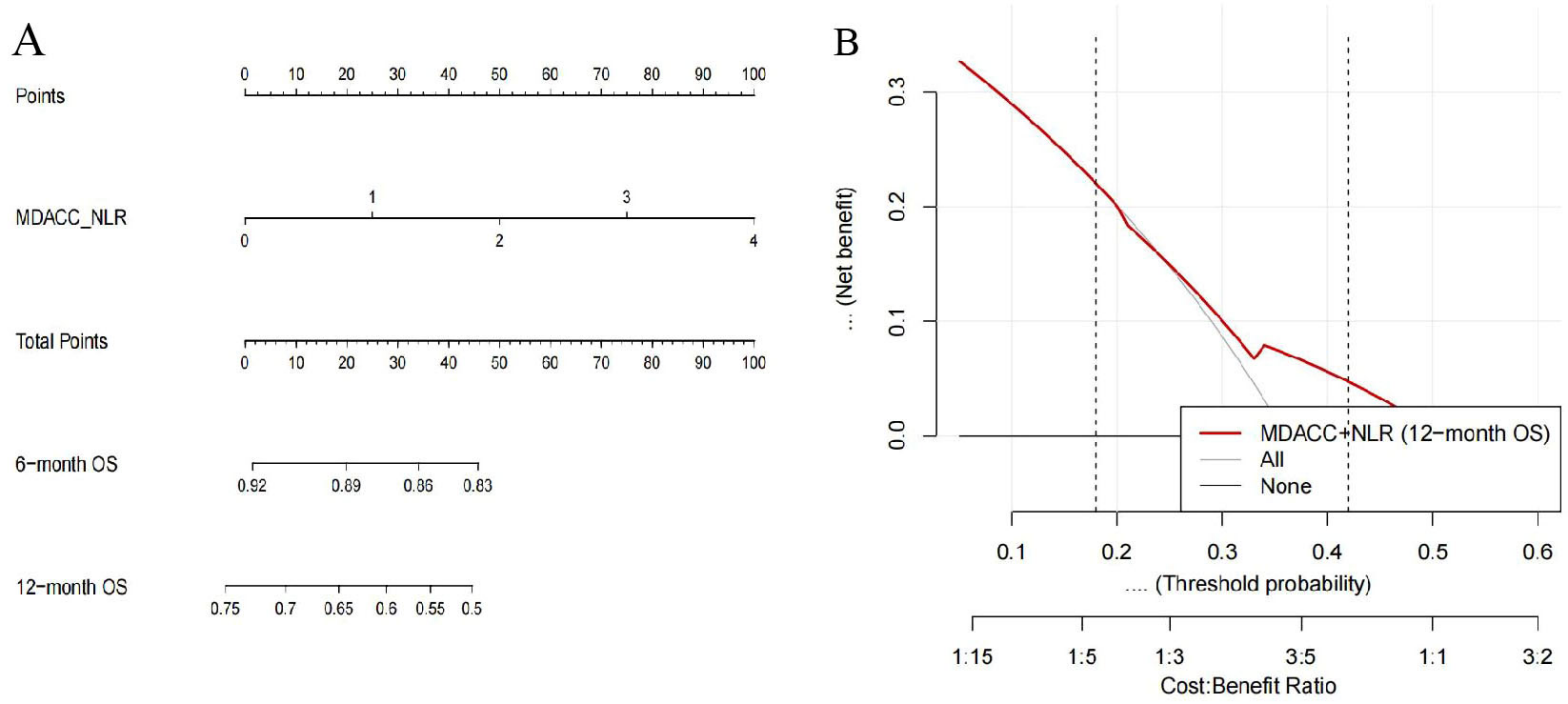
Nomogram and decision curve analysis (DCA) based on the MDACC+NLR scoring system for predicting overall survival (OS) in extensive-stage small-cell lung cancer (ES-SCLC) patients receiving first-line chemoimmunotherapy. **(A)** Nomogram developed from the MDACC+NLR score using a Cox proportional hazards model to predict 6- and 12-month OS. The upper “Points” axis assigns a score to each MDACC+NLR value; summing these yields the “Total Points,” which can be projected downward to estimate the predicted 6- and 12-month OS probabilities. The MDACC+NLR axis is displayed over a range of 0–4 for visualization, where 3–4 represent extrapolated ranges beyond the observed data (observed range = 0–2). **(B)** Decision curve analysis (DCA) evaluating the clinical net benefit of the MDACC+NLR-based model for 12-month OS. The red curve indicates the MDACC+NLR model, and the gray/black lines represent the reference “treat-all” and “treat-none” strategies. The model demonstrates a positive net benefit within threshold probabilities of approximately 0.18–0.42, supporting its potential clinical utility.

The MDACC + NLR–based Cox model demonstrated significant prognostic value for overall survival (OS) in patients with extensive-stage small-cell lung cancer (ES-SCLC) receiving first-line chemoimmunotherapy. The apparent Harrell’s concordance index (C-index) for OS was 0.62, and the bootstrap-corrected C-index was 0.61, indicating moderate discrimination. Calibration curves at 6 and 12 months showed good agreement between predicted and observed survival probabilities. The corresponding nomogram (**Figure 8A**) provides individualized estimates of 6- and 12-month OS probabilities according to the MDACC + NLR score. Decision curve analysis (DCA) (**Figure 8B**) revealed a positive net benefit across clinically relevant threshold probabilities of approximately 0.18–0.42, exceeding the “treat-all” and “treat-none” strategies. These findings confirm the satisfactory predictive accuracy and potential clinical utility of the MDACC + NLR model for prognostic assessment in ES-SCLC.

## 4 Discussion

### 4.1 Clinical implications

Small cell lung cancer (SCLC) is an aggressive malignancy classified as limited-stage (LS) or extensive-stage (ES), with ES-SCLC accounting for 70%–80% of cases and characterized by distant metastases and poor prognosis (17). Platinum-based chemotherapy has long been the first-line standard, achieving limited efficacy (median OS <12 months) (18). The advent of chemoimmunotherapy (CIT) has improved survival outcomes.

To date, few studies have systematically assessed immune–inflammation–based prognostic models such as NLR in SCLC. In this real-world cohort, the MDACC+NLR scoring system demonstrated the best predictive performance among all evaluated models. From a translational perspective, the MDACC+NLR model offers a pragmatic and easily applicable tool for risk stratification and individualized treatment planning in the immunotherapy era (19).

Although several prognostic models—such as LIPI, GRIm, RHM, and MDA-ICI—have shown prognostic value in NSCLC, none demonstrated significant discrimination in our ES-SCLC cohort (20–22). This discrepancy likely reflects the unique biological characteristics of SCLC, including rapid proliferation, genomic instability, and a neuroendocrine yet immunologically “cold” phenotype with low PD-L1 expression and limited T-cell infiltration. Consequently, inflammation-based indices originally derived from NSCLC fail to capture the distinct immune dysfunction of SCLC, and their risk cutoffs (e.g., for NLR or LDH) may not be directly applicable. These mechanistic and biological differences may underlie why only the MDACC+NLR model retained robust predictive performance in ES-SCLC (23).

Based on this framework, we further validated the prognostic performance of these models in our real-world ES-SCLC cohort (24). In our cohort, the MDACC+NLR model demonstrated the strongest predictive ability, with scores ≤1 associated with longer PFS and OS. The number of metastatic organs, also confirmed as an independent prognostic factor (25–27), may further enhance MDACC+NLR performance.

Landmark trials—IMpower133, CASPIAN, and KEYNOTE-604—have confirmed the survival benefit of CIT in ES-SCLC (10, 11, 18). IMpower133 demonstrated OS and PFS improvement with atezolizumab plus chemotherapy (11), CASPIAN verified similar results with durvalumab (10), and KEYNOTE-604 supported pembrolizumab in this setting (18). Consistent with these pivotal studies, our real-world analysis of 197 patients showed longer PFS (8 vs. 6 months; HR = 0.63; P < 0.01) and OS (15 vs. 13 months; HR = 0.71; P < 0.01) in the CIT group, confirming the effectiveness of CIT outside trial settings.

Multivariate Cox analysis identified ECOG ≥1, lung metastases, and liver metastases as independent adverse factors for both PFS and OS, consistent with prior evidence^[23–25]^. ECOG status reflects treatment tolerance and prognosis, while liver metastases are known to promote an immunosuppressive microenvironment impairing ICI efficacy. Although some HRs included 1 within their confidence intervals, these trends warrant validation in larger cohorts.

Effective prognostic tools should provide clear survival discrimination using accessible parameters. The MDACC+NLR system fulfills these criteria and represents a valuable, pragmatic model for prognosis and treatment planning. Previous studies have only explored MDACC+NLR in small or exploratory SCLC cohorts (19); our large-scale, real-world validation provides robust evidence to support its integration into future risk models and clinical practice.

### 4.2 Inflammation-based biomarkers

Peripheral blood biomarkers such as ANC, NLR, dNLR, PLT, and LDH are cost-effective indicators of systemic inflammation and immunotherapy response (28–31). Elevated levels correlate with poor outcomes, and recent studies confirm that high baseline inflammatory scores predict reduced response and shorter survival in SCLC patients receiving CIT (32). These easily accessible markers thus offer practical value for early risk stratification.

### 4.3 Biomarker perspective in SCLC

To date, the NCCN Clinical Practice Guidelines for small cell lung cancer (SCLC) have not recommended routine testing for PD-L1 expression or tumor mutational burden (TMB), due to insufficient evidence linking these biomarkers with clinical benefit from immunotherapy. Although PD-L1 and TMB are well-established predictive biomarkers in non-small cell lung cancer (NSCLC), their clinical utility in SCLC remains limited because of low PD-L1 expression, methodological heterogeneity, and restricted tissue availability.

In the CASPIAN trial, PD-L1 positivity was observed in approximately 5.7% of tumor cells and 25.8% of immune cells (28.3% in either), and durvalumab plus chemotherapy improved survival regardless of PD-L1 status (11). Similarly, the IMpower133 analysis showed that atezolizumab plus chemotherapy prolonged overall survival independently of PD-L1 and other biomarker subgroups (10). Consequently, the NCCN SCLC guideline does not currently recommend routine PD-L1 or TMB testing. These findings emphasize that, in contrast to molecularly driven strategies in NSCLC, SCLC still lacks validated biomarkers for immunotherapy selection—highlighting the rationale for exploring immune-inflammatory scoring model.

Similarly, tumor mutational burden (TMB) has been investigated as a potential biomarker for predicting the efficacy of immunotherapy in SCLC. The 2025 CSCO Clinical Practice Guidelines for Small Cell Lung Cancer indicate that TMB may serve as a predictive indicator of response to immune checkpoint inhibitors, and that NGS-based multigene panel testing represents a clinically feasible approach for estimating TMB (33). In the phase I/II CheckMate 032 trial, patients with high TMB who received nivolumab plus ipilimumab achieved an objective response rate (ORR) of 46.2% and a 1-year progression-free survival (PFS) rate of 30.0%, which were significantly higher than those in the low- and intermediate-TMB subgroups (34). Furthermore, when tumor tissue is insufficient, NGS-based circulating tumor DNA (ctDNA) testing is considered a promising alternative method for TMB evaluation (35, 36). Despite these encouraging results, TMB testing is currently classified as a Grade III recommendation, Level 2B evidence in the CSCO guidelines, and neither NCCN nor CSCO currently recommend routine TMB testing in SCLC. The predictive value of TMB in this disease still requires validation in larger prospective studies.

In our cohort, 197 patients were included, of whom only 11 had available PD-L1 testing data. Among these, 8 patients showed PD-L1 <1%, and 3 patients showed PD-L1 = 0%, consistent with previous studies reporting generally low PD-L1 expression in SCLC. In addition, TMB testing was not performed because comprehensive genomic profiling was not part of the standard diagnostic workflow during the study period. These findings align with current NCCN and CSCO recommendations, which do not support routine PD-L1 or TMB testing in SCLC.

Given these limitations, our study further highlights the clinical importance of developing immune–inflammation–based prognostic models, such as the MDACC+NLR scoring system, which utilize readily accessible clinical and hematological parameters. These models may provide a practical and cost-effective approach for prognostic stratification in real-world settings, complementing molecular biomarker research and bridging the gap between biological insight and clinical applicability.

### 4.4 Limitations and future directions

This study has several limitations. First, its retrospective, single-center design may introduce inherent selection bias. Second, the absence of an external validation cohort limits the generalizability of our findings. Third, molecular biomarkers such as PD-L1 expression and tumor mutational burden (TMB) were not routinely assessed, as these tests are not yet mandated by current clinical guidelines and remain relatively costly in real-world practice. Future prospective studies incorporating molecular and immune biomarkers are needed to confirm and expand these findings. Fourth, as this was a retrospective study, data on treatment-related adverse events (AEs) were incomplete, which precluded analysis of the potential association between AEs and prognostic scores. Future multicenter, prospective studies incorporating external validation, molecular biomarker analyses, and safety outcomes are warranted to confirm and extend our findings. Future multicenter prospective studies integrating molecular and immune biomarkers, external validation, and treatment toxicity analysis are warranted to refine and validate the MDACC+NLR model. In future work, we plan to further enlarge the dataset and integrate additional biological correlates to enhance the generalizability and mechanistic insight of our findings (37).

### 4.5 Conclusion

This study demonstrates that poor performance status (ECOG ≥1), lung metastasis, and liver metastasis are independent adverse prognostic factors for both progression-free survival (PFS) and overall survival (OS) in patients with extensive-stage small cell lung cancer (ES-SCLC) receiving first-line chemoimmunotherapy. Among the prognostic models evaluated, the MDACC+NLR scoring system exhibited superior predictive accuracy for treatment outcomes and survival, suggesting its potential utility as a valuable tool to guide personalized therapeutic strategies in ES-SCLC.

## Data Availability

The original contributions presented in the study are included in the article/**Supplementary Material**. Further inquiries can be directed to the corresponding author.
