# Supplementary material for: Prognostic value of the MDACC–NLR score in extensive-stage small-cell lung cancer treated with first-line chemoimmunotherapy
Source: Front Immunol. 2025 Nov 7;16:1681658. doi: 10.3389/fimmu.2025.1681658 (PMC12634505; doi:10.3389/fimmu.2025.1681658)
Supplement: Supplementary file 1 [file Table1.docx]

**Appendix Table 1 Summary of Tumor Immunotherapy-Related Prognostic Scoring Systems**

| Rating system | Scoring indicators | Scoring criteria | Risk grouping |
| --- | --- | --- | --- |
| RMH | LDH >245U/L（ULN）  ALB<35g/L  Number of transferred organs>2 | 1 point will be awarded for each abnormality | 0-1 point: Low risk  2-3 points: High risk |
| MDACC | Number of transferred organs>2  ECOG ≥1  LDH>ULN  ALB<35g/L  Gastrointestinal tumor type=Yes | 1 point will be awarded for each abnormality | 0-1 point: Low risk  2 points: Medium risk  3-5 points: High risk |
| MDACC+NLR | Number of transferred organs>2  ECOG ≥1  LDH>ULN  ALB<35g/L  Gastrointestinal tumor type=Yes  NLR＞6 | 1 point will be awarded for each abnormality | 0-1 point: Low risk  ≥ 2 points: High risk |
| MDA+ICI | Age>52 years old  Liver metastasis=present  ECOG>1  ANC>4.9x109/L  ALC<1.8x109/L  PLT＞300 ×109/L  LDH＞183 | 1 point will be awarded for each abnormality | 0-2 points: Low risk  3 points: Medium risk  4 points: medium to high risk  5-7 points: High risk |
| LIPI | dNLR >3  LDH>ULN | 1 point will be awarded for each abnormality | 0 points: Low risk  1 point: Medium risk  2 points: High risk |
| GRIm | NLR >6  LDH>ULN  ALB <35g/L | 1 point will be awarded for each abnormality | ≤ 1 point: Low risk  >1 point: High risk |

RMH: Royal Marsden Hospital; MDACC: MD Anderson Cancer Center; NLR: Neutrophil-to-Lymphocyte Ratio; ICI: Immune Checkpoint Inhibitor; LIPI: Lung Immune Prognostic Index; LDH: Lactate Dehydrogenase; ALB: Albumin; PS: Performance Status (ECOG score); ANC: Absolute Neutrophil Count; WBC: White Blood Cell count; dNLR: Derived Neutrophil-to-Lymphocyte Ratio; CRP: C-Reactive Protein
